# European NCAP Program Developments to Address Driver Distraction, Drowsiness and Sudden Sickness

**DOI:** 10.3389/fnrgo.2021.786674

**Published:** 2021-11-19

**Authors:** Rikard Fredriksson, Michael G. Lenné, Sjef van Montfort, Colin Grover

**Affiliations:** ^1^Swedish Transport Administration, Skövde, Sweden; ^2^Department of Mechanics and Maritime Sciences, Chalmers University of Technology, Göteborg, Sweden; ^3^European New Car Assessment Programme (Euro NCAP), Leuven, Belgium; ^4^Monash University Accident Research Centre, Monash University, Melbourne, VIC, Australia; ^5^Seeing Machines, Canberra, ACT, Australia; ^6^TNO, Integrated Vehicle Safety Department, Helmond, Netherlands; ^7^Thatcham Research, Berkshire, United Kingdom

**Keywords:** distraction, drowsiness, driver monitoring, test protocols, consumer testing, NCAP, vehicle safety, road safety

## Abstract

Driver distraction and drowsiness remain significant contributors to death and serious injury on our roads and are long standing issues in road safety strategies around the world. With developments in automotive technology, including driver monitoring, there are now more options available for automotive manufactures to mitigate risks associated with driver state. Such developments in Occupant Status Monitoring (OSM) are being incorporated into the European New Car Assessment Programme (Euro NCAP) Safety Assist protocols. The requirements for OSM technologies are discussed along two dimensions: detection difficulty and behavioral complexity. More capable solutions will be able to provide higher levels of system availability, being the proportion of time a system could provide protection to the driver, and will be able to capture a greater proportion of complex real-word driver behavior. The testing approach could initially propose testing using both a dossier of evidence provided by the Original Equipment Manufacturer (OEM) alongside selected use of track testing. More capable systems will not rely only on warning strategies but will also include intervention strategies when a driver is not attentive. The roadmap for future OSM protocol development could consider a range of known and emerging safety risks including driving while intoxicated by alcohol or drugs, cognitive distraction, and the driver engagement requirements for supervision and take-over performance with assisted and automated driving features.

## The Need for Occupant Status Monitoring

Driver distraction and drowsiness remain significant contributors to death and serious injury on roads around the world. Recent data from Europe and Australia confirm that approximately 25% of crashes involve drowsiness, and that distraction and inattention are factors in 29–48% of fatal and serious injury crashes (Sundfør et al., 2019; Fitzharris et al., 2020; European Commission, 2021a). In 2019 in the United States nearly 4,000 fatalities (11% of the total) and over 400,000 injuries were attributed to distraction or drowsiness (NHTSA, 2020, 2021). These numbers are likely to be underestimates given the difficulty of identifying crash causation with these factors. Sudden sickness is also a common cause of fatal crashes. In around 10% of fatal crashes in Sweden, and 6% of injury crashes in Australia, the driver suddenly became severely ill and lost control of the car (Fitzharris et al., 2020; Trafikanalys, 2021).

Road crashes attributed to distraction and drowsiness are long-standing issues in road safety strategies around the world. Road safety countermeasures have educated the public to the dangers of impaired driving and improved road infrastructure and occupant protection. Today there is even greater competition for a driver's attention. Competition for attention stems from external influences such as an increasingly busy, urbanized traffic environment and roadside (dynamic) advertising, alongside the proliferation of personal mobile devices and the “always on” society.

Managing risks in real time associated with distraction and drowsiness, as is done by intelligent speed adaptation for speeding behavior for example, has historically not been technologically possible. There is much research available now that supports the use of direct monitoring approaches, such as camera-based OSM, and that has informed the development of European Commission regulations mandating this type of technology in future years (Hynd et al., 2015). Apart from the obvious use of driver monitoring cameras to detect distraction and drowsiness, indirect symptoms of sudden sickness and driving under influence (DUI) can also be captured (e.g., head falling down or drowsiness) by the same technology and create an added benefit for these areas (Lenné, 2021).

## European NCAP Roadmap and Objectives

Each year the European New Car Assessment Programme (Euro NCAP) tests all new high volume selling car models (>90% of cars sold have a rating) to provide consumer information regarding the overall safety of these cars. A total star rating is based on four areas: Adult occupant, Child occupant, Vulnerable road user and Safety assist. Protocols are typically updated every 2 years to increase the safety level. Major changes to these are laid out in a roadmap every 5 years. Under the current Euro NCAP roadmap (Euro NCAP, 2017) direct driver monitoring will be required from 2023 onwards to get a full score in the Occupant Status Monitoring (OSM) area as part of the Safety Assist Protocol. Providing a warning to drivers is important, however a stronger safety benefit will be seen if OSM is integrated with ADAS such that ADAS can become more sensitive if the driver is showing signs of inattention, drowsiness or sudden sickness. It is an important complement to the already existing areas of passive and active protection and driver support in areas such as Speed Assist systems.

## Dimensions of OSM Capability

Euro NCAP's objective is to provide a strong safety outcome without over trust and an acceptable user experience to support consumer acceptance. This requires thinking about the behaviors that will be captured under the Euro NCAP program and setting definitions and test scenarios that support the stated objectives. There are two key dimensions to understanding OSM capability: detection difficulty and behavioral complexity.

The ability to detect and track the driver reliably in more complex environments equates to system availability and the proportion of time a system could provide protection to the driver. A less capable technology might be able to track in constant and less challenging environmental conditions seen in a driving simulator laboratory, but performance would degrade markedly in variable and bright lighting conditions experienced regularly in on-road driving. Particular aspects of driver appearance can also challenge performance that include eye shape and skin texture along with the driver seating position (typically indicated by driver height). Increased capability on this dimension is evident by high levels of detection accuracy with a wider range of “noise factors” that include sunglasses, hats, and masks for example.

The more recent academic and industry focus has been on defining the behaviors linked to increased risk and in developing solutions to address them. The simplest and most well-understood type of distraction behavior is a single long glance away from the roadway and is associated with increased crash risk (Victor et al., 2015). However, not all distraction meets this simplistic behavioral definition. More complex distraction behaviors are evident when drivers engage in secondary tasks such as phone use while driving. Drivers often engage in visual time sharing, where attention is split between driving and a secondary task, often up to 20–30 s (Lenné et al., 2020). This concept is recognized in several published distraction models (Seppelt et al., 2017; Kircher et al., 2020), and is important to capture to maximize safety outcomes.

**Figure 1:**
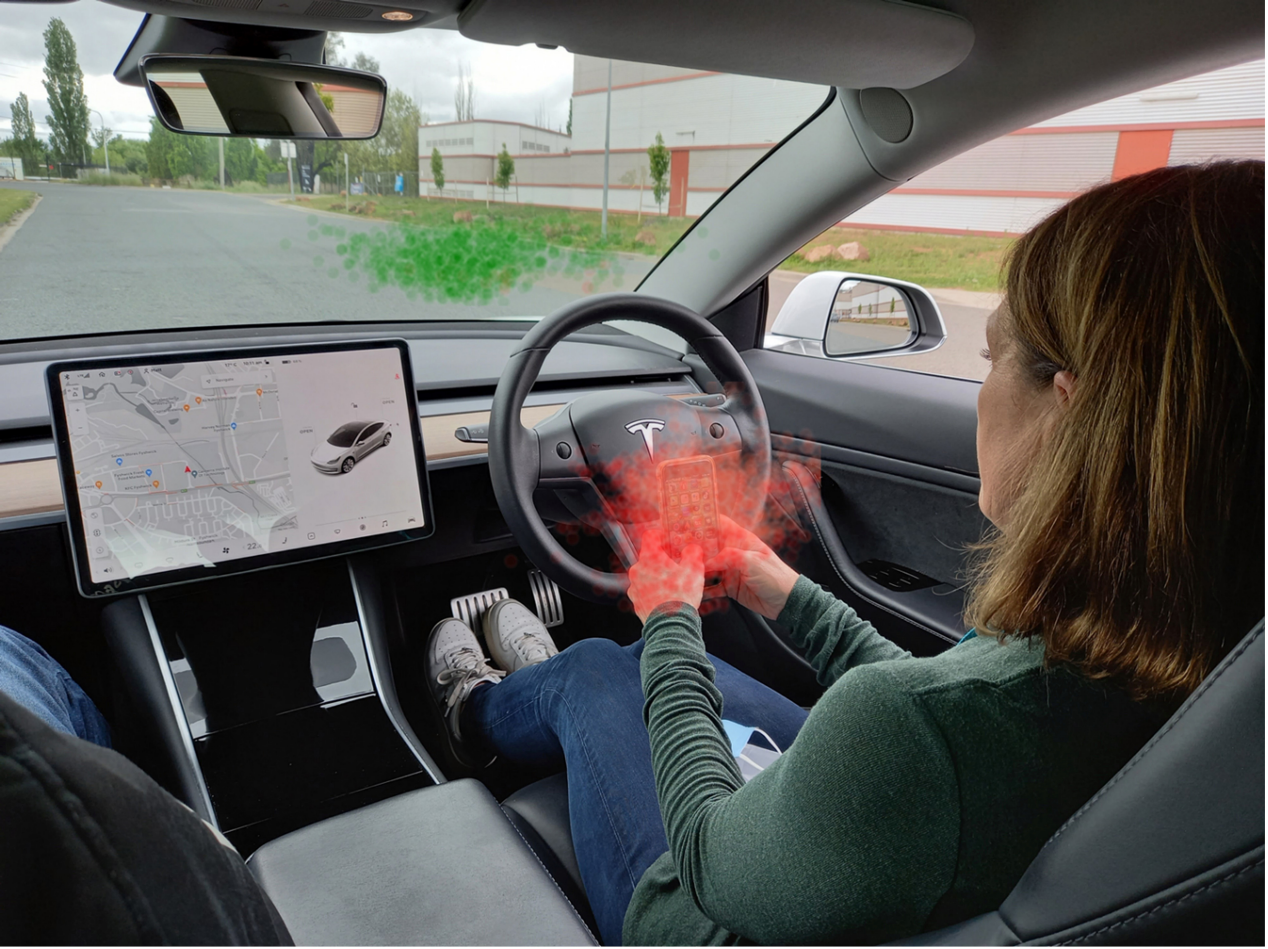
Example of a driver using of a mobile phone with a driver assistance feature. Indicative metrics are presented for eye gaze (red) and head pose (green) when a driver uses a lizard glance strategy while looking at mobile phone.

The movement of a driver's head and eyes is also important. For glances that are a smaller visual angle from the forward roadway drivers typically will engage in what is termed “lizard” glance behavior. Here the drivers' eyes are moving but the head is relatively still (Fridman et al., 2016). In contrast, for glances to areas that are larger visual angle from the forward roadway, regions such as the side window and passenger seat, drivers typically engage in an “owl” strategy, where the shifting of visual attention is primarily achieved by head rotation followed by the eyes. Figure 1 illustrates lizard visual behavior while using a phone and presents both eye gaze and head pose orientation for those sequences where the driver is looking at the phone (adapted from Yang et al., 2021). The drivers head pose remains orientated on-road. Only detection using eye gaze would detect this example of phone use. Detecting cell phone distraction will be significantly improved with approaches that measure visual behavior directly through eye gaze metrics rather than relying on head pose alone or indirect measures.

Drowsiness-related behaviors can also be characterized through a similar lens of increasing complexity. Simplistic measures of drowsiness may only capture eyelid behavior or indirect measurements. PERCLOS is an example of an eyelid-based metric used to establish a drowsy state (typically over 20 mins), however its performance is modest (Sommer and Golz, 2010; Jackson et al., 2016). Individual variability in drowsiness progression and symptoms mean that systems that rely on single drowsiness metrics are insufficient to capture drowsiness reliably (Ingre et al., 2006; Chua et al., 2014). Multiple signs of drowsiness, including blink duration, amplitude-velocity ratio and frequency and are likely to capture more patterns of drowsiness behavior (Caffier et al., 2003; Lee et al., 2016; Liang et al., 2019). The lack of defined objective drowsiness measures presents some additional challenges to those faced in monitoring distraction.

Microsleeps are included in the protocol, where a microsleep is a momentary period of sleep where the driver is unconscious. Microsleeps have traditionally been defined through Electroencephalography (EEG), with intrusions of theta waves anywhere between 3 and 15 s (Liang et al., 2019; Hertig-Godeschalk et al., 2020). EEG defined microsleeps have been linked with driver impairment and crash risk (Boyle et al., 2008; Golz et al., 2011). Microsleep identification through EEG is currently both impractical in driving and limited by the temporal capabilities and signal noise of the technology. Increasingly, behavioral characteristics of microsleeps have been linked to physiological and performance indicators of severe drowsiness, with long eye closures being the primary visual indicator of a microsleep (Buckley et al., 2016; Mulhall et al., 2020). In its simplest form an OSM detected microsleep could be triggered by a long eye closure, with eye closures >500 ms linked to measures of driver risk (Alvaro et al., 2016; Mulhall et al., 2020). However, there are a range of behaviors such as yawning and squinting that could be mis-interpreted as drowsiness-related long eye closure events. A simplistic definition would therefore produce a higher number of false alerts and would not provide high levels of driver acceptance. More complex definitions of microsleeps, such as those that accommodate additional indicators of microsleep (e.g., head nodding) or evidence of prior drowsiness, are needed to ensure that drivers are not receiving an excessive level of false alarms and to provide the intended safety benefits.

Recognizing sudden sickness is also part of the protocol and presents a unique challenge to data collection and ecological validity. Sudden sickness can be used as an umbrella term covering a variety of conditions (e.g., diabetic shock, cardiac events, seizures, etc.), where the common result is driver incapacitation. These events are unpredictable by nature, resulting in very sparse data, and therefore there is currently no method or taxonomy to detail these categories and their related behavior. It is reasonable to assume, however, that the driver is neither performing driving tasks effectively nor responding to vehicle alerts. In the early stages of implementation it is therefore reasonable to regard sudden sickness as a period of lack of response which can be implemented as an escalation of either drowsiness or distraction which goes uncorrected.

**Figure 2:**
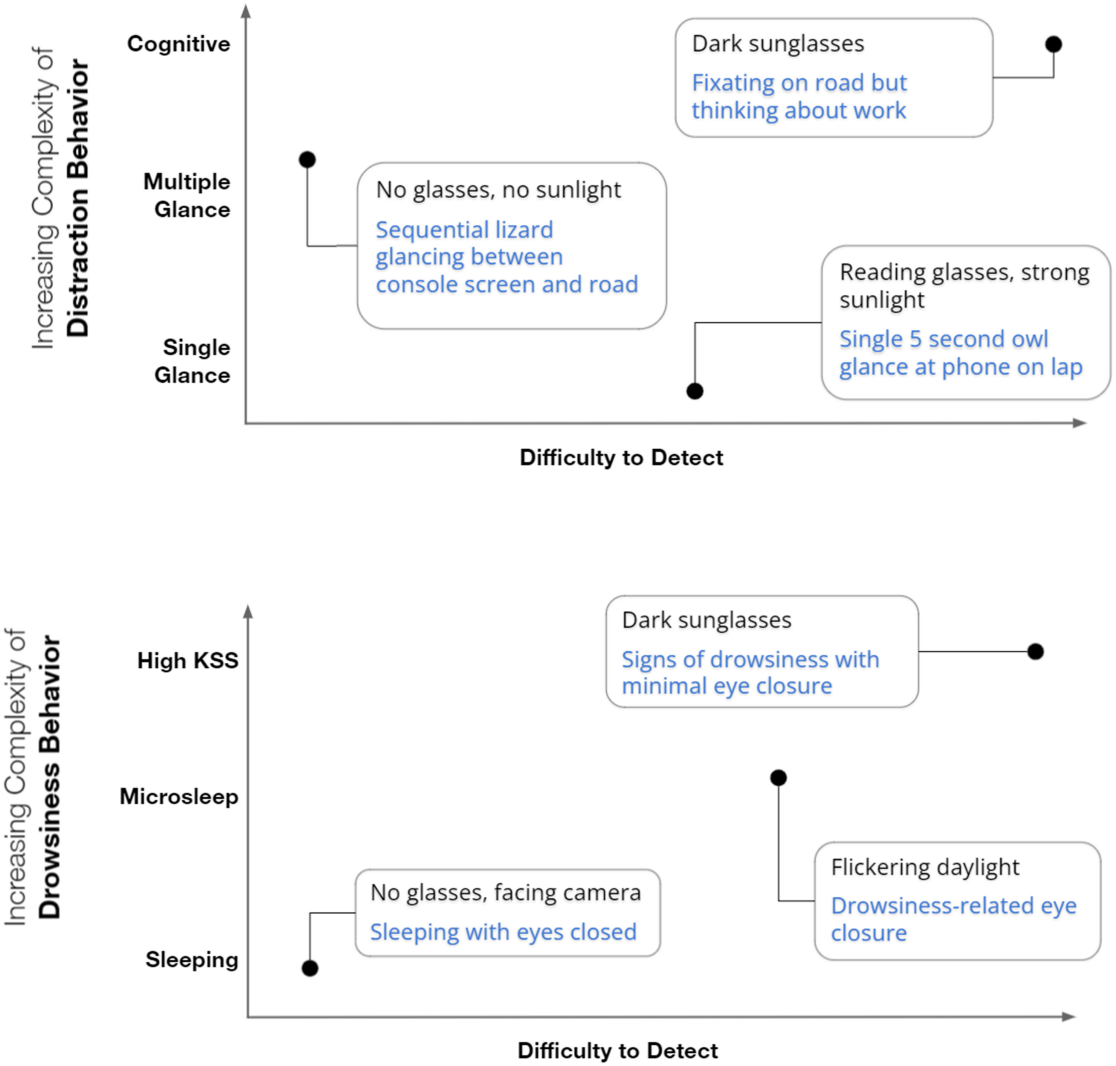
The behavior-technology matrix for distraction (top) and drowsiness (bottom) that describes different levels of camera-based OSM technology performance. Within each figure the blue text represents the complexity of driver behavior, while the black text represents noise variables that could alter detection difficulty.

The behavior-detection matrix differentiates performance based upon the projected level of protection to the driver (Figure 2). A simpler detection technology (left end of *x* axis) with simpler behavioral features (bottom end of *y* axis) will only be able to reliably perform in the bottom left corner of the matrix. A more sophisticated technology with robust behavioral features will be able to perform toward the top right of the matrix, therefore providing coverage over a much greater range of scenarios and representing a superior solution. This matrix provides the basis of the range of noise variables and behaviors that are covered in the proposed protocol.

## Test Methodology

It is important that the protocol finds a balance that provides a safe and acceptable outcome for the community while implementing processes that are manageable by OEMs. It should incentivize widespread adoption while still affording opportunities for differentiation. It should encourage the implementation of systems that are not simply pure warning systems to the driver, but go further to integrate the OSM signal with other ADAS systems. Making ADAS systems such as automatic emergency braking or lane keep systems more sensitive when a driver is distracted, for example, is expected to provide both a greater safety benefit and more acceptable driver experience.

Protocol development considers a range of driver appearance and noise factors to ensure acceptable levels of system availability and thus system performance. The approach here is to test systems across the extremes of driver appearance, for example tall through to short drivers, and drivers of different ages, very young through to very old. Collecting data across these factors ensures good system availability with a wide range of seating positions and skin textures (wrinkles, baggy eyes). These appearance variables can be described very precisely, as is routinely done in published research studies, to give clear guidance to OEMs on the conditions being tested. The same philosophy holds with introducing noise factors into the testing.

Testing behaviors are the second element of the matrix presented in Figure 2. For distraction these behaviors include: single long glances to specified driving-related and non-driving-related targets, and; visual time sharing behaviors (multiple short glances) that address risks associated with engagement in secondary activities including phone use. A test example for visual time sharing tasks could include scripted glance sequences from on-road to the console over a 10–15 s period. Testing toward the extremes of the owl and lizard glance strategies separately is a key element. This ensures that a range of individual differences in glance strategies are accommodated while also accommodating a key element that can differentiate the capability of an OSM feature. Distraction scenarios will need to be tightly prescribed and highly repeatable. Testing drowsiness-related behaviors is somewhat more complex as no single behavior or pattern is consistent across all individuals (Caffier et al., 2003; Chua et al., 2014). This makes reproducing drowsiness behaviors in a consistent manner problematic. Drowsy and microsleep data should therefore be collected from drivers that are genuinely drowsy and where this can be confirmed by validated measures [e.g., the Karolinska Sleepiness Scale (KSS) or EEG].

Ideally all testing would be conducted in test track conditions as is done with existing Euro NCAP AEB/Lane Support protocols. Track testing with a sufficient number of drivers, with different appearance, incorporating different noise factors, and testing across the range of distraction and drowsiness behaviors is not practical. The approach initially proposes testing using a dossier of evidence provided by the OEM alongside selected use of track testing. The dossier approach provides guidance to OEMs without being overly prescriptive and limiting advancements in early stage technologies, and may include recommendations of best practice guidelines for testing drowsiness, such as number of subjects and methods of inducing and validating drowsiness. Deviations from guidelines will require supporting evidence justifying the method and demonstrating comparable performance and safety benefits of the alternate approach.

Performance assessment is a key part of any testing methodology. The test philosophy of Euro NCAP is to assess how well a safety system works when needed (true positives), while the false alarm rate (false positives) is assigned to the vehicle manufacturer to address. Publicly available data for distraction algorithms estimate sensitivity performance exceeding 80% however false positives can exceed 20% (Lee et al., 2013). For drowsiness, current General Safety Regulation standards for legal acceptance are understood to place sensitivity around 40%; this mark is achievable by several algorithms but bears room for improvement at false-positive rates of 11–24% (Friedrichs and Yang, 2010; Anderson and Horne, 2013). Simply put, a vehicle with unacceptable false alarm rate will not provide an acceptable customer experience. The requirements for appropriate driver warning and vehicle intervention are directly linked to both safety outcomes and driver experience and should ensure an appropriate balance is struck between sensitivity and specificity.

## Future Directions in Sensing and Testing

The roadmap for future protocol development could consider a broader spectrum of behaviors and states linked to driver impairment. Alcohol and other drugs are examples given the links to fatal crashes in Europe (25% of all fatalities are alcohol-related; European Commission, 2021b), and the documented potential for real-time OSM approaches (Lenné et al., 2020; Hayley et al., 2021). We noted earlier the need for research efforts to shed new light on related features such as sudden sickness to further enhance their utility over time.

Insights from widespread implementation are likely to provide new insights for warning and intervention strategies. For drowsiness in particular combining performance and behavioral indicators, such as steering and ocular inputs for example, may improve prediction performance. From a warning and intervention viewpoint there is acknowledgment that drowsiness alerts alone will get us so far and that additional intervention strategies are needed to improve safety outcomes in the long term (Fitzharris et al., 2017). The full integration of OSM into the suite of ADAS affords an expanded range of real-time vehicle intervention strategies.

While risks associated with distraction and single long glances away from the forward roadway are well-understood, further research is needed on safety impacts of multiple glance distraction. For example: at what point during a given sequence does a driver become distracted; how is this influenced by the driver's engagement in driving and non-driving related tasks; how does the external environment influence this; and what are the links to probable crash types? Further, cognitive distraction and inattention are emerging safety issues. While reasonably well-understood in driving simulator studies, direct links to real world safety are less well-documented. Crash types here include “looked but failed to see” where a driver's visual attention can be directed on-road yet they are cognitively engaged in another activity.

Current Safety Assist protocols are designed to support drivers operating vehicles in manual driving, i.e., without assisted or automated driving functions. Driver behavior will change with increases in driver assistance and vehicle automation as drivers increasingly have the opportunity to take hands off wheel and/or eyes off road under defined conditions. It is critical to consider what safety issues these changes might introduce and how OSM can best support safe outcomes. Driver engagement is the cognitive state that is increasingly important to understand and measure here from a safety perspective (Lenné et al., 2020). Drivers need to remain sufficiently engaged and attentive to the driving task to ensure they are able to resume control should the assistance feature not perform as expected. It its simplest form, if a driver is known to be sufficiently attentive, this knowledge could be used to allow ACC to proceed from a stand-still at a red light, for example. Driver take-over readiness is key as it informs take-over performance, a safety outcome included in the planning for future Euro NCAP protocols.

There are several opportunities for researchers and industry to pursue to close some knowledge gaps. For researchers, perhaps it is about establishing the safety risks and safety scenarios for driver states that are less understood, such as cognitive distraction. Conducting in-depth crash studies to better understand the crash types and associated driver behaviors and system factors—helping to set the agenda for the problems that both technology development and safety policy should target. Continued research into the most effective warning and intervention strategies is also key. For industry there is an immediate opportunity to combine with other sensors such as child presence detection, seat belt wearing detection (advanced SBR), and occupant position and size for in-crash protection systems. There is also the opportunity to continue to push the boundaries on the safety cases that can be addressed, and the underlying technologies used to achieve this, to ensure that even greater injury reductions are realized.

## Conclusion

Distracted and drowsy driving are highlighted as key sources of road trauma in road safety strategies around the world. These behaviors have historically been very difficult to identify when they occur while driving. OSM technologies offer new opportunities to manage driver distraction and drowsiness in real-time and thus reduce fatal and serious injury. We believe this is best achieved by combing warning and intervention strategies such as, for example, increasing the sensitivity of driver assistance systems when a driver is not attentive. The European NCAP continues to evolve its OSM protocols to recognize more advanced technologies such as driver monitoring as an integral part of upcoming rating protocols that will reward vehicle manufacturers who provide OSM features in future vehicles.

Protocols have been developed that attempt to address and mitigate the higher risk distraction and drowsiness behaviors. These protocols are likely to become effective for new vehicle models in Europe from 2023 and evolved for a 2025 update. The European NCAP roadmap in the future could include a number of known and emerging safety issues that could include cognitive distraction and take-over performance.

## Data Availability Statement

The original contributions presented in the study are included in the article/supplementary material, further inquiries can be directed to the corresponding author.

## Ethics Statement

Written informed consent was obtained from the individual(s) for the publication of any potentially identifiable images or data included in this article.

## Author Contributions

RF and ML: conceptualization. ML led preparation of the original draft with RF. RF and ML: writing, reviewing, and editing. SvM and CG assisted with writing, reviewing and editing. All authors approved the final manuscript as submitted.

## Conflict of Interest

ML was employed by and holds shares in Seeing Machines. The remaining authors declare that the research was conducted in the absence of any commercial or financial relationships that could be construed as a potential conflict of interest.

## Publisher's Note

All claims expressed in this article are solely those of the authors and do not necessarily represent those of their affiliated organizations, or those of the publisher, the editors and the reviewers. Any product that may be evaluated in this article, or claim that may be made by its manufacturer, is not guaranteed or endorsed by the publisher.
